# *Lactiplantibacillus plantarum* and *Saussurea costus* as Therapeutic Agents against a Diabetic Rat Model—Approaches to Investigate Pharmacophore Modeling of Human IkB Kinase and Molecular Interaction with Dehydrocostus Lactone of *Saussurea costus*

**DOI:** 10.3390/metabo13060764

**Published:** 2023-06-19

**Authors:** Metab A. AlGeffari, Dina Mansour, Omar Ahmed-Farid, Einas Mohamed Yousef, Shereen A. Mohamed, Mahmoud M. A. Moustafa, Hassan Barakat, Khalid Abd El Ghany

**Affiliations:** 1 Department of Family and Community Medicine, College of Medicine, Qassim University, Buraydah 51452, Saudi Arabia; m.geffari@qu.edu.sa; 2 Diabetes Center, Medical City, Qassim University, Buraydah 51452, Saudi Arabia; 3 Pharmacology Department, Medical Research and Clinical Studies Institute, National Research Centre, Giza 12622, Egypt; 4 Department of Pharmacology and Toxicology, Faculty of Pharmacy, Galala University, Attaka, Suez 43511, Egypt; 5 Department of Physiology, Egyptian Drug Authority, EL-Manial, Cairo 11511, Egypt; 6 Department of Histology and Cell Biology, Faculty of Medicine, Menoufia University, Shibin el Kom 32511, Egypt; 7 Department of Genetics and Genetic Engineering, Faculty of Agriculture, Benha University, Moshtohor 13736, Egypt; 8 Department of Food Science and Human Nutrition, College of Agriculture and Veterinary Medicine, Qassim University, Buraydah 51452, Saudi Arabia; 9 Food Technology Department, Faculty of Agriculture, Benha University, Moshtohor 13736, Egypt; 10 Department of Microbiology, Egyptian Drug Authority, Cairo 11511, Egypt

**Keywords:** *Lactiplantibacillus plantarum*, *Saussurea costus*, 16S rRNA gene, biochemical, histological analysis, pharmacophore modeling, docking, diabetes mellitus

## Abstract

Lactic acid bacteria is well-known as a vital strategy to alleviate or prevent diabetes. Similarly, the plant *Saussurea costus* (Falc) Lipsch is a preventive power against diabetes. Here, we aimed to determine whether lactic acid bacteria or *Saussurea costus* is more effective in treating a diabetic rat model in a comparative study manner. An in vivo experiment was conducted to test the therapeutic activity of *Lactiplantibacillus plantarum* (MW719476.1) and *S. costus* plants against an alloxan-induced diabetic rat model. Molecular, biochemical, and histological analyses were investigated to evaluate the therapeutic characteristics of different treatments. The high dose of *S. costus* revealed the best downregulated expression for the *IKBKB*, *IKBKG*, *NfkB1*, *IL-17A*, *IL-6*, *IL-17F*, *IL-1β*, *TNF-α*, *TRAF6*, and *MAPK* genes compared to *Lactiplantibacillus plantarum* and the control groups. The downregulation of *IKBKB* by *S. costus* could be attributed to dehydrocostus lactone as an active compound with proposed antidiabetic activity. So, we performed another pharmacophore modeling analysis to test the possible interaction between human IkB kinase beta protein and dehydrocostus lactone as an antidiabetic drug. Molecular docking and MD simulation data confirmed the interaction between human IkB kinase beta protein and dehydrocostus lactone as a possible drug. The target genes are important in regulating type 2 diabetes mellitus signaling, lipid and atherosclerosis signaling, NF-κB signaling, and IL-17 signaling pathways. In conclusion, the *S. costus* plant could be a promising source of novel therapeutic agents for treating diabetes and its complications. Dehydrocostus lactone caused the ameliorative effect of *S. costus* by its interaction with human IkB kinase beta protein. Further, future studies could be conducted to find the clinical efficacy of dehydrocostus lactone.

## 1. Introduction

Diabetes is a worldwide public health threat with adverse impacts on humans, healthcare systems, and societies. According to the International Diabetes Federation, 537 million individuals endured diabetes mellitus in 2021. This number is expected to rise to 643 million by 2030 and 783 million by 2045 [1]. Diabetes is a chronic endocrine, metabolic disease characterized by an insufficient or resistant insulin response [2]. It is a complex condition for all metabolic problems that result in chronic hyperglycemia, driven by interactions between genetic, environmental, lifestyle, and socioeconomic factors [3]. Since ancient times, medicinal plants have been focused on as essential agents in diabetic treatments. Traditional treatments commonly employed *S. costus* as a medicinal plant with a wide spectrum of bioactivities, such as hypolipidemic, diuretic, antimicrobial, anti-inflammatory, and antioxidant properties [4,5]. Recent research has evaluated this plant’s antidiabetic potential [6,7]. The phytochemical profile of *Saussurea costus* evidenced the presence of carbohydrates, triterpenoids, proteins, alkaloids, tannins, saponins, flavonoids, and steroids as active ingredients [8], which contributes to the diverse qualities [9].

Recently, lactic acid bacteria have been attracting considerable interest for safe and effective treatment of diabetic treatments [10]. Lactic acid bacteria can maintain the ideal combination of gut flora by interacting with their metabolites and producing different antibacterial substances such as organic acids, diacetyl, and hydrogen peroxide [11]. *Lactiplantibacillus plantarum* strains have been shown in animal studies and clinical trials to lower blood lipids and cholesterol as a proposed treatment for diabetes [12]. They can lower blood pressure by using these active ingredients generated mostly from antihypertensive peptides and peptidoglycan to improve the immune system’s general functioning [13]. 

IkappaB kinase (IKK) is a protein complex regulated by type 2 diabetes. It is involved in the NF-_K_B signaling pathway [13]. The IKK complex is made up of three subunits: IKK-alpha (IKK1), IKK-beta (IKK2), and IKK-gamma (IKK3) (NEMO) [14]. The alpha and beta subunits have the catalytic activity of the IKK complex, while the gamma subunit regulates it. IKBKB gene expression is critical for gene regulation in various signaling pathways, including IKBKB type 2 diabetes and MAPK signaling [15].

Hence, lactic acid bacteria and *S. costus* have promising antidiabetic effects, but which is more effective with the highest one? To answer this question, the current work compared *L. plantarum* and *S. costus* as two strategies for treating alloxan-induced diabetic rats by molecular, biochemical, and histological examinations. Another pharmacophore modeling approach of human IkB kinase as a diabetic regulation factor was performed to prove its molecular interaction with the dehydrocostus lactone of *S. costus*, revealing the best antidiabetic effects. Gene regulation of several stress pathways and primarily essential genes implicated in type 2 diabetes was also investigated. 

## 2. Materials and Methods

### 2.1. Materials

All chemicals and reagents were purchased from Sigma-Aldrich, St. Louis, MO, USA. 

### 2.2. S. costus Collection and Extraction

*S. costus* dry roots were bought from Cairo’s national herbal shop. For three days, the powder root of *S. costus* (500 g) was steeped in 2500 mL of 70% ethanol. The supernatant was purified using Whatman No. 1 filter paper and collected using a rotary evaporator at 45 °C before being kept at 4 °C for future research [16].

### 2.3. LC–MASS Spectrometry Analysis of S. costus Extract

An *S. costus* root extract was spun at 16,000 rpm for ten minutes at 4 °C before filtering via a 0.22 µm filtering system. The prepared samples were inserted into the Agilent 1290 system’s autosampler using the method Gheraibia, and his coworkers used with minimal adjustments [17].

### 2.4. Sample Collecting, Bacterial Isolation, and Identification

A hundred-gram white cheese pattern was diluted (1:10) using an MRS broth medium (HiMedia Laboratories, Thane (West), India), homogenized nicely in a stomacher bag, stamped, and incubated in a single day at 30 °C to enhance lactobacilli. Samples of 0.1 mL were plated overnight on a pour MRS agar and incubated anaerobically (5% CO_2_) at 37 °C. After forty-eight hours, bodily distinct and nicely separated colonies had been picked and streaked onto new His MRS agar plates. Colonies with lactobacillus-like characteristics were randomly selected and enriched in MRS broth. The isolation method was repeated until *Lactobacillus* was acquired. The gross appearance of all colonies was tested for cultural and morphological features. Colony length, form, color, and texture have all been recorded. Lactic acid isolates were described using the techniques Zhao et al. used [18]. 

### 2.5. Bacterial Genomic DNA Purification

The bacterial culture of the current isolate was harvested at the mid-log phase by centrifuging 5 mL at 8000 rpm for six min and discarding the aqueous solution. The bacterial pellet was vortexed with a 400 μL TE buffer and 100 μL 5 M NaCl with 1 mg mL^−1^ lysozyme that was incubated at 37 °C for 2 h. Additionally, after one hour at 60 °C, 0.5 mg mL^−1^ proteinase K was added to completely lyse the bacterial mixture. Then, 50 µL of 10% CTAB was joined and stood at 70 °C for 1 h with occasional mixing by inverting the tube. Next, we centrifuged the cell lysate in a microcentrifuge at 12,000 rpm for 10 min. Proteins were extracted twice with equal volumes of a chloroform:isoamyl (24:1) solution. The aqueous solution was moved to a sterilized 1.5 mL vial. An equal volume of icy absolute HPLC grade 2-propanol was added to the genomic DNA. The DNA pellet was cleaned twice with 1.5 mL of 70% HPLC-grade ethanol. The dried DNA pellet was resuspended in 100 µL of a TE buffer and stocked at −20 °C for future studies. DNA concentration and purity were measured using a BioTek Epoch 2 spectrophotometer at 260/280 nm (BioTek, now Agilent Company, 5301 Stevens Creek Blvd Santa Clara, CA 95051, United States). DNA was migrated through a 1.2% agarose gel and stained with EtBr. A Gel Doc XR+ System visualized DNA. A 1 Kb DNA ladder (Promega Corporation, 2800 Woods Hollow Road Madison, WI 53711, USA) was used as a marker, as described by Mahmoud et al. [19].

### 2.6. PCR Recipe and Program

Primer3Plus [20] was utilized to generate specific 16S rRNA gene primers for *L. plantarum.* The primers’ efficacy and specificity were examined utilizing *Insilico* PCR online tool [21]. A 50 µL combined volume consisted of 5 µL 10X PCR buffer, 5 µL 2X dNTPs mixture (0.4 mM each), 1 µL 10 pmol of each primer (16S rRNA, Table 1), 0.2 µL Taq DNA polymerase (Premix TaqTM, TaKaRa TaqTM Version 2.0, Code No. R004A), 1 µL 50 ng DNA template, and 37.8 µL free RNase water to complete the reaction volume that underwent thermal cycling in a Thermocycler (Labcycler Gradient 96 block, SensoQuest, Hannah-Vogt-Str. 1, 37085, Göttingen, Germany). The amplification conditions included 4 min at 94 °C before commencing thirty-five cycles at a 1 min denaturation at 94 °C followed by sixty-second annealing at 60 °C, and elongation lasted for another minute at 72 °C and concluded with an additional 10 min extension step running again at 72 °C. Upon completion, the amplicon was stained with an EtBr in 1X TAE buffer after being run on a gel containing 1.5% agarose and was monitored via a Gel Doc XR + System afterward. As a marker, a 100 bp ladder (Promega, UK) was used, as described by Mahmoud et al. [19].

### 2.7. Molecular Identification

The PCR segment was refined via the QIAquick PCR Purification Kit, as specified by the manufacturer. Macrogen Company, located in South Korea, conducted the sequencing of this purified amplicon. A 16S rRNA sequence alignment and comparison were made against sequences presented in NCBI’s nucleotide database with the BLASTn algorithm to identify closely related bacteria. Phylogenetic tree construction utilized the maximum likelihood method based on the Tamura-Nei model implemented in MEGA ver. 11 software to display the evolutional relationship between isolates, and those found closest in the database [19].

### 2.8. Cells Lysis

HiMedia Laboratories, India, cultured *L. plantarum* F1031 in ten liters of MRS broth and incubated it at 37 °C for 18 h. Cells were then collected by centrifugation at 8000 rpm for 10 min. After three PBS washes, the cells were disturbed with a microfluidizer at a pressure of 10–13,000 psi and a temperature of 3–10 °C. Many disruption procedures (up to 9) may have been necessary to ensure complete bacterial lysis. Following freeze-drying, the lysate is formed and packed in a controlled environment, such as a sterilized cabinet [22].

### 2.9. Animals

Adult male Sprague Dawley rats weighing 150 and 170 g were obtained from the animal house run by the Egyptian drug authority (EDA). All animals were kept and treated in conformity with standards set forth by the EDA’s animal care and use committee under ethical number NODCAR/13/4/2020. During the acclimation period, before the beginning of the trials, the animals were housed in normal conditions and provided with regular food and water. Diabetes was symptomatically induced before the experiment via intraperitoneal injections of alloxan monohydrate (A7413-10G, CAS No. 2244–11–3, Sigma-Aldrich St. Louis, MO, USA) at a dose of 170 mg kg^−1^ body weight diluted in normal saline. Glucose levels exceeding 140 mg dL^−1^ indicated hyperglycemia resulting from the treatment modified by the method described by Nakamura et al. [23].

### 2.10. Experimental Design

Six separate groups of five rats were studied. Group one acted as the control and was labeled G1. The other five groups, labeled 2–6, were induced with diabetes through a single dose (170 mg kg^−1^ BW) with an intraperitoneal injection of alloxan dissolved in a 0.01 M citrate anhydrous buffer at pH 4.5. The diabetic group of five rats was separated and labeled G2 after the induction. These diabetic rats had three additional treatments: intact cells made up of *L. plantarum* (1 × 10^9^ CFU mL^−1^ daily); lysate cells (1 × 10^9^ CFU mL^−1^ daily) from *L. plantarum*; and a low dose (25 mg kg^−1^ BW) of *S. costus* and a high dose (100 mg kg^−1^ BW) of *S. costus* make up groups 3–6, respectively. During the first two weeks, bacterial and botanical sources were introduced daily for all groups except the control and diabetic group as prophylactic treatments. Alloxan was injected on day 14, and for the confirmation of diabetes induction, the animals were left without treatment on days 15 and 16. After day 16, bacterial and botanical sources were introduced again daily as treatments for diabetes. Diabetes was detected by recording blood glucose concentrations 72 h after alloxan injection and was modified by Imtiaz et al. [24].

### 2.11. Body Weight and Body Weight Gain 

Body weight was determined according to Rolland et al. [25]. Body weight gain was calculated using the method described by Bravo et al. [26]. 

### 2.12. Sample Collection and Preparation

After 30 days, male Sprague Dawley rats were sacrificed. The liver, spleen, pancreas, lung, and kidney organs were removed and washed with normal saline before being frozen in liquid nitrogen and kept at −80 °C. Frozen tissues were then weighed and lyophilized in liquid nitrogen, as previously described. Blood samples were taken during tissue harvesting, and the portion was centrifuged at 4 °C for 15 min. The blood samples were collected in serum gel tubes for further examination. Tissue powder was extracted with 6% PCA and neutralized with 6 M potassium hydroxide (KOH). The aqueous solution was refined through a 0.45 μm filter for glutathione analysis.

### 2.13. Biochemical Parameters

As indicated in previous investigations, hydrolyzed and derivatized tissue samples were utilized to assess malondialdehyde (MDA) levels, as described in [27]. Nuhu’s team developed an HPLC technique for analyzing samples for GSH and GSSG levels [28]. Using a BioTek Epoch 2 reader, blood samples from fasting rats were examined for total cholesterol (TC), triglyceride (TG), high-density lipoprotein (HDL), and low-density lipoprotein (LDL) [29]. Using the stated procedure, plasma samples from fasting rats were obtained and utilized to measure glucose and insulin levels [30]. As previously demonstrated, the HOMA-IR was computed by multiplying fasting glucose (mg dL^−1^) by fasting insulin (pmol L^−1^) and dividing the result by 22.5 [31].

### 2.14. Histological Study

After scarification, livers were removed and kept in 10% buffered formaldehyde for microscopic inspection. All test individuals in each experimental group received blocks of these livers preserved by formalin-fixed paraffin embedding. Bancroft et al. [32] described how to fix and stain sections (5 μm thick) using Hematoxylin and Eosin (H&E). Two researchers independently evaluated pathology grades for H&E-stained slides using light microscopy and the Ishak-modified histology activity index system [27]. The grading method was as follows: 1 for no anomaly, 2 for minor changes (10%), 3 for moderate changes (25%), 4 for significant changes (50%), and 5 for severe/marked alterations (>75%). Images were captured with an Olympus SC30 digital camera and an Olympus BX-46 microscope.

### 2.15. Total RNA Extraction

TissueLyser II (Qiagen, Hilden, Germany) was used to homogenize 50 mg of unfrozen tissue from each sample within a 2 mL tube containing 700 μL GENEzolTM Reagent (GENEzol^TM^ TriRNA Pure Kit, GZX050, GZXD050). The homogenized material was spun at 14,000 rpm for one minute before being transferred to a 2 mL RNase-free tube. An equal volume of absolute ethanol (molecular grade) was added to this mixture and properly mixed before inserting the RB column in a separate 2 mL collection tube. This combination was spun at 14,000 rpm for one minute, with the followthrough removed after that. This process was repeated until the RB column was complete before transferring it to another sterilized 2 mL vial, adding 50 μL or freshly prepared DNase I to its center, and then pre-washing it with a 400 μL pre-wash buffer, which was also spun at 14,000 rpm for one minute before discarding the resultant follow through the material. To clean the column, it was centrifuged for three minutes. After being eluted with 50 μL RNase-free water, the RNA was immediately kept at −80 °C for the subsequent RT-PCR procedure described by Brunt [33].

### 2.16. cDNA Synthesis

The 20 μL cDNA reaction was made in two steps. The first required incubating a PCR machine with 10 μL total RNA, a 2 μL oligo (dt) 18 primer, and 1.5 μL RNase-free water for 10 min (SensoQuest, Hilden, Germany). In the second stage, 4 μL of a 5X first strand buffer, 0.5 μL of H minus MMLV (200 unit/μL), and 2 μL of dNTPs mixture (10 mM) were blended, added to the first step, and kept for 60 min at 42 °C. The initial cDNA samples were kept at −80 °C for the subsequent qPCR process, as described by Badr et al. [34].

### 2.17. Gene Expression

Quantitative PCR experiments were performed three times. The PCR reaction included 10 μL of mater mix (A.B.T.TM 2X qPCR SYBR-Green MasterMix, ROX, Q03-02-01), 0.4 μL ROX Dye (50X), 1 μL of each primer (10 μM) listed in Table 1, a 150 ng cDNA template, and up to 20 μL of UltraPure H2O. The qRT-PCR procedure is described as follows. The first denaturation cycle lasted 5 min at 95 °C. Forty cycles were conducted at 95 degrees Celsius for 30 s and 60 degrees Celsius for 45 s. The melting slope lasted 5 s at 80 degrees Celsius. The two housekeeping genes (GAPDH and β-actin) were employed as the internal controls. Because the β-actin factor displayed a more robust representation, it was used to normalize qRT-PCR data. The gene expression of the goal mRNA was figured to be close to a reference gene for each specimen (∆Ct sample = Ct target gene − Ct reference gene). Gene expression transcripts were given as a normalization ratio of fold = 2^−∆∆Ct^ sample [35].

**Table 1:** The primers list used in this study.

Primer	Sequence	References
16S rRNA	F: 5′-TTTGAGTGAGTGGCGAACTG-3′ R: 5′-TTCATGTAGGCGAGTTGCAG-3′	This study
GAPDH	F: 5′-AAGCTGGTCATCAATGGGAAAC-3′ R: 5′-ACCCCATTTGATGTTAGCGG-3′	[36]
β-actin	F: 5′-CGGAACCGCTCATTGCC-3′ R: 5′-ACCCACACTGTGCCCATCTA-3′	[36]
IKBKB	F: 5′-AGGGTGGTTTTTTATTTTTATTTT-3′ R: 5′-AACCCCCACTAAAACTAACTTAA-3′	[37]
IKBKG	F: 5′-GGCCAAACAGGAGGTGAT-3´ R: 5′-TTCTTCTCGGCCAGCTTC-3´	[38]
NfkB1	F: 5´-CCTGTCTGAAGCCCTGCTACA-3´ R: 5´-CACACCCTGGTTCAGAAGCTG-3′	[39]
IL-17A	F: 5′-CTTCACCCTGGACTCTGAGC-3′ R: 5′-TGGCGGACAATAGAGGAAAC-3′	[40]
IL-6	F: 5′-ACCACCCACAACAGACCAGT-3′ R: 5′-CAGAATTGCCATTGCACAAC-3′	[41]
IL-17F	F: 5′-TCTTCAATCAAAACCAGGGCAT-3′ R: 5′-GGAGTTCATGGAGCCGTCTT-3′	[42]
IL-1β	F: 5′-AAATGCCTCGTGCTGTCTGA-3′ R: 5′-AGGCCACAGGGATTTTGTCG-3′	[43]
TNF-α	F: 5′-CCCAGACCCTCACACTCAGATCAT-3′ R: 5′-GCAGCCTTGTCCCTTGAAGAGAA-3′	[44]
TRAF6	F: 5′-CTCAGCGCTGTGCAAACTAC-3′ R: 5′-GATCAAGGATCGTGAGGCGT-3′	[45]
MAPK	F: 5′-TGATATTTGGTCTGTGGGCTG-3′ R: 5′-TGTTCCACGGCACCTTATTTT-3′	[42]

### 2.18. Molecular Docking 

#### 2.18.1. Screening of Ligand Properties and ADMET Prediction

Lipinski’s five-parameter rule was used to assess all of the compounds. Lipinski’s rule was offered through the SwissADME web tool at http://www.swissadme.ch/index.php, accessed on 14 May 2023. The ProTox-II online program (https://tox-new.charite.de/protoxII/index.php?site=home accessed on 14 May 2023) was utilized to anticipate toxicity risks. The SMILES of the ligand molecules retrieved from https://pubchem.ncbi.nlm.nih.gov accessed on 14 May 2023 were uploaded into the online tool.

#### 2.18.2. Preparation of the Receptor and Drug 

The target protein (4KIK) was obtained from the RCSB Protein Data Bank website, found at https://www.rcsb.org, accessed on 14 May 2023. The ligands were obtained from https://pubchem.ncbi.nlm.nih.gov, accessed on 14 May 2023 as 3D SDF files. UCSF-Chimera version 1.17.1 generated the receptor protein and ligand (dehydrocostus lactone, 73174). The protein was produced by removing all unnecessary residues and then going to Dockprep. Autodock vina identified the grid box coordinates and sizes for the pocket site (protein–ligand interaction site). The pdb-prepared receptor and ligand mol2 files were uploaded to UCSF-Chimera version 1.17.1, and Autodock vina was run.

#### 2.18.3. Multiple Molecular Docking 

The dehydrocostus lactone similarity ligands were collected from http://www.swisssimilarity.ch, accessed on 14 May 2023. Dockprep was used to prepare the previous receptor.pdpqt file for multiple molecule docking. Open Babel version 3.1.1 was used for ligand minimization and conversion. Multiple docking was accomplished using the Perl program Vina Linux.pl. The high-affinity kcal/mol docking findings were visualized using UCSF-Chimera version 1.16. Maestro software version 2022-4 and Discovery Studio version 2021 were used to visualize the 2D ligand–protein interaction.

#### 2.18.4. Molecular Dynamic Simulation 

The optimal affinity posture of the target (4KIK) and ligand was produced using Dockprep on UCSF-Chimera version 1.17.1 and was saved as a PDB file without the ligand. The ligand was reduced and saved as a mol2 file. Swissparam.ch was used to create the ligand mol2 file. LIG.zip was downloaded and unzipped. The molecular dynamic simulation was carried out using GROMACS version 2022.4 and GROMACS tutorial 5: Protein-Ligand Complex [46]. The MD simulation results were shown using Grace 5.1.25.

### 2.19. Statistical Analysis

The results were represented as the mean ± SE for each group of six rats. SAS’s one-way variance analysis was utilized to determine group variations (ANOVA). The data gathered was statistically analyzed using the general linear model (GLM). Duncan’s Multiple Range Test was performed to see if there were any statistically significant changes in means. Differences were judged significant when the *p*-values were less than 0.05.

## 3. Results

### 3.1. Biochemical and Molecular Identification

**Figure 1:**
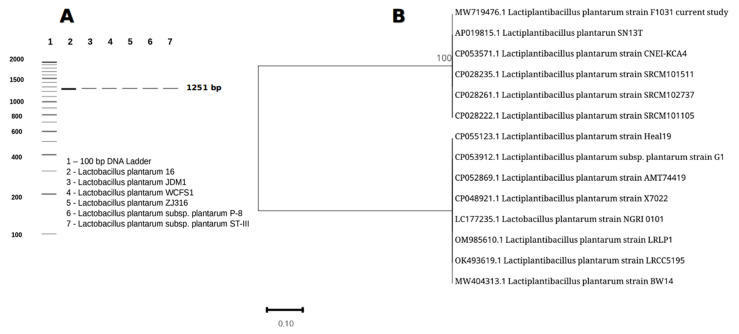
(**A**) In silico PCR of the current primer sequences matched with *L. plantarum* strains with an amplicon of 1251 bp, http://insilico.ehu.es/ accessed on 5 December 2022. (**B**) The created phylogenetic tree showed our current isolate with the nearest ones registered in the NCBI database.

With an identity ratio of 89.6%, the biochemical identification of the bacterial isolate indicated that the present isolate belonged to *L. plantarum*. The data on the efficiency and sensitivity of the 16S rRNA gene primers validated with *L. plantarum* strains were provided in Figure 1A. The anticipated in silico amplicon size was 1251 bp. The assembled contig sequence was 1511 bp long and was entered into the GenBank database. In NCBI GenBank, the obtained sequence has accession number MW719476.1. As shown in Figure 1B, the alignment analysis using the BLASTn program revealed that the closest match for the current isolate was *L. plantarum* (AP019815.1), with a 100% identity ratio. The phylogenetic tree also validated the link between the present bacterial isolate (MW719476.1) and the other *L. plantarum* strains in the same cluster.

### 3.2. LC Mass Analysis

**Figure 2:**
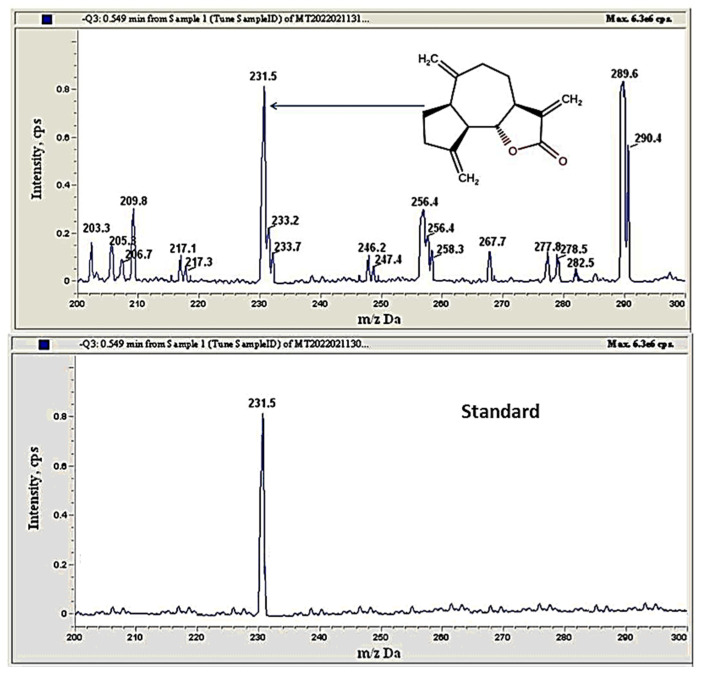
LC MASS analysis showed the peak (231.5 m/z Da) of the chemical compound of dehydrocostus lactone, C15H18O2, from the extracted roots of *S. costus*.

The peak of dehydrocostus lactone was detected in the LC MASS findings as a secondary metabolite of *S. costus*. As shown in Figure 2, the retention period of the emerging dehydrocostus lactone standard was comparable to the extract of dry roots of *S. costus*.

### 3.3. Growth Performance Parameters

Table 2 indicated that the diabetes group had a significantly lower ultimate body weight than the control group. The treated groups with intact cells, lysate cells, *S. costus* 25 mg kg^−1^ BW (low dose), and *S. costus* 100 mg kg^−1^ BW (high dose) all exhibited a substantial gain in body weight with the same sequence as the diabetic group. Otherwise, the High dose performed improved oxidative stress markers (Table 3), lipid profile (Table 4), glucose, and insulin levels more than intact cells, lysate cells, and the low dose (Table 5). 

### 3.4. Biochemical Parameters

The diabetic group had considerably greater MDA, GSSG, and lower GSH than the control group, according to the results in Table 3. Furthermore, compared to the control group, diabetes groups treated with intact cells, lysate cells, low dosage, and high dosage dramatically reduced oxidative stress indicators. Unlike the diabetic group, the treated groups with intact cells, lysate cells, and low and high doses all exhibited a substantial increase in GSH with the same sequence. Otherwise, there were substantial differences between the greatly improved groups of high doses, low doses, lysate cells, and intact cells. The diabetic group diminished all measures except MDA, whereas the treated groups showed increased levels of oxidative stress markers compared to the diabetic group.

According to the findings in Table 4, the diabetic group had considerably higher TG, TC, and LDL levels and lower levels of HDL than the control group. Furthermore, diabetic groups treated with intact cells, lysate cells, and a low dosage dramatically exhibited better lipid profile levels than the diabetic group. These groups exhibited a substantial drop in TC, TG, and LDL and a rise in HDL compared with the diabetic group. 

The findings in Table 5 indicated that the diabetic group had a substantial rise in glucose and a reduction in insulin compared to the control group. Furthermore, compared to the control group, diabetic groups treated with intact cells, lysate cells, low dosage, and high dosage revealed improved diabetes indicators. Compared to the diabetic group, the treated group with intact cells, lysate cells, and a modest dosage increased insulin significantly with the same sequence. Otherwise, there were substantial differences between the greatly improved groups of high doses, low doses, lysate cells, and intact cells. The diabetic group administrated with a high dose of *S. costus* exhibited increased insulin levels compared to the diabetic group.

### 3.5. Histological Examinations

**Figure 3:**
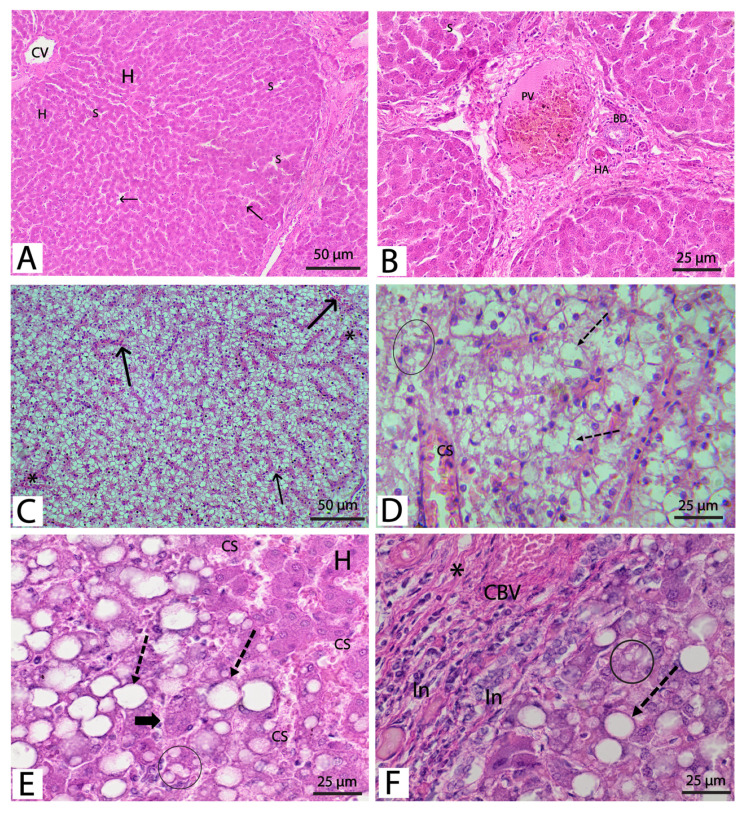
Micrograph of the H&E-stained sections of liver tissues of the (**A**) control group showing a normal histological structure of the liver with irregular plates of hepatocytes (H) radiating from the central vein (CV), separated by vascular sinusoids (S). (**B**) The control group shows the portal tract located at the peripheral part of the hepatic lobules, which includes a venule (a branch of the portal vein) (PV), an arteriole (a branch of the hepatic artery) (HA), and a bile ductule (BD). (**C**) A diabetic rat (alloxan-treated groups) demonstrates considerable disruption of hepatic architecture with generalized fatty change in hepatocytes, which gives the cytoplasm a foamy appearance. (**D**) A diabetic rat demonstrates most hepatocytes with peripheral nuclei; few hepatocytes show microvesicular steatosis (black circle). Notice the congested sinusoid (CS) and no clear lobular pattern. (**E**,**F**) A diabetic rat showing combined microvesicular (black circle) and macrovesicular (dotted arrows) fatty change, while a few hepatocytes appear normal (H), a few enlarged hepatocytes with peripheral flattened nuclei (thick black arrow), prominent periportal fibrous tissue (*) infiltrated with inflammatory cells (In). Notice the congested hepatic sinusoids (CS) and the congested blood vessels (CBV). (Stain: H&E; A, C: 100×, B, D, E, F: 400×).

The control group’s H&E-stained slides revealed the typical microscopic architecture of the liver, including characteristic hepatic lobules with central veins. Hepatocytes were polyhedral acidophilic cells with solitary vesicular nuclei; nevertheless, some cells were binucleated and arranged as cords spreading from the central vein. Hepatic sinusoids separated hepatocytes, tiny crevices between hepatocyte cords bordered by flattened endothelial cells, and a few Kupffer cells (Figure 3A). The portal tracts revealed stroma, including terminal branches of the portal vein, a branch of the hepatic artery, and a bile duct in the peripheral region of the hepatic lobules (Figure 3B). Light microscopy of H&E-stained liver slices from alloxan-treated rats revealed substantial disruption of the typical concentric arrangement of hepatocytes and portal arteries and the absence of the hepatic lobular pattern. The hepatic sinusoids were found to be dilatable and congested to varying degrees. Most hepatocytes displayed microvesicular and macrovesicular fatty degeneration, with tiny and large steatotic vacuoles forming inside the cells. Some hepatocytes seem pale with eccentric or flattened nuclei, others display hypertrophy, and others appear normal. Almost all hepatocytes display substantial fatty alteration in some places, making their cytoplasm have a foamy appearance. Significant connective tissues exist between the hepatic lobules and interlobular and periportal leucocytic infiltration (steatohepatitis) (Figure 3C–F). Pathological alterations in diabetic rats were documented in more than 75% of the tissues investigated (Table 3).

**Figure 4:**
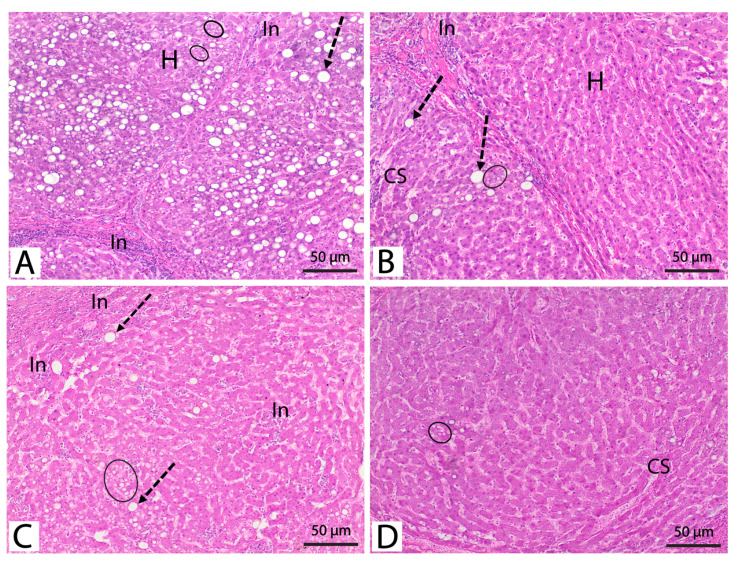
Micrograph of the H&E-stained sections of the liver tissues of (**A**) diabetic rats treated with *L. plantarum* cell lysate showing hepatocytes with microvesicular (black circle) and macrovesicular steatosis (dotted arrow), normal hepatocytes (H), and inflammatory infiltration (steatohepatitis) (In). (**B**) The diabetic rats treated with *L. plantarum* intact cells showed normal morphology in most hepatocytes (H), a few hepatocytes showing large fat vacuoles (dotted arrow), as well as a few hepatocytes showing microvesicular fatty changes (black circle). Notice the congested hepatic sinusoids (CS) and the inflammatory infiltrate (In). (**C**) The diabetic rats treated with a low dose of *S. costus* show that most hepatocytes seem normal, some appear vacuolated with small steatotic vacuoles (black circle), and very few hepatocytes exhibit large steatotic vacuoles. Notice the inflammatory cell infiltration (In) between hepatocytes and the portal area’s connective tissue. (**D**) The diabetic rats treated with a high dosage of *S. costus* showed normal hepatic architecture; some hepatocytes exhibited microvesicular fatty change (black circle) and a few congested hepatic sinusoids (CS) (Stain: H&E; 100×).

The hepatic microscopic architecture of diabetic rats treated with *L. plantarum* cell lysate improved somewhat compared to the alloxan-treated group. Despite micro and macrovesicular steatosis in many hepatocytes being found, the number of unaffected hepatocytes increased. In addition, inflammatory infiltrations and fibrosis persisted, albeit to a smaller level than in the diabetic untreated group (Figure 4A). Notably, the revealed pathological abnormalities in this group received a four since degenerative alterations were observed in around 50% of the tissues examined (Table 2). Tiny slices from diabetic rats treated with *L. plantarum* intact cells revealed a significant improvement in alloxan-induced liver degradation compared to the diabetic group. Most hepatocytes appear normal; nevertheless, some hepatocytes had microvesicular fatty degeneration, and only a few more hepatocytes exhibited macrovesicular fatty degeneration. In addition, there was evidence of obstructed hepatic sinusoids and inflammatory infiltration (Figure 4B). Obviously, this group fared better since degenerative changes were found in around 25% of the analyzed liver tissue (Table 6).

Compared to the alloxan-treated group, H&E-stained sections of the diabetic rats treated with a low dose of *S. costus* (25 mg kg^−1^) demonstrated a significant improvement in the histological architecture of the liver. Most hepatocytes look normal; however, some hepatocytes appear vacuolated with tiny steatotic vacuoles, and a few hepatocytes have large steatotic vacuoles. Inflammatory cell infiltration was found between cell plates and the portal connective tissue (Figure 4C). Furthermore, the histological architecture of hepatic tissue from diabetic rats given a high dose of *S. costus* was identical to the normal controls. However, few hepatocytes displayed microscopic steatotic vacuoles, and a few congested hepatic sinusoids were found in a few scattered regions (Figure 4D). Sections taken from rats treated with a low and high dosage of *S. costus* were scored as three and two, respectively. (Table 6).

### 3.6. Gene Expression 

**Figure 5:**
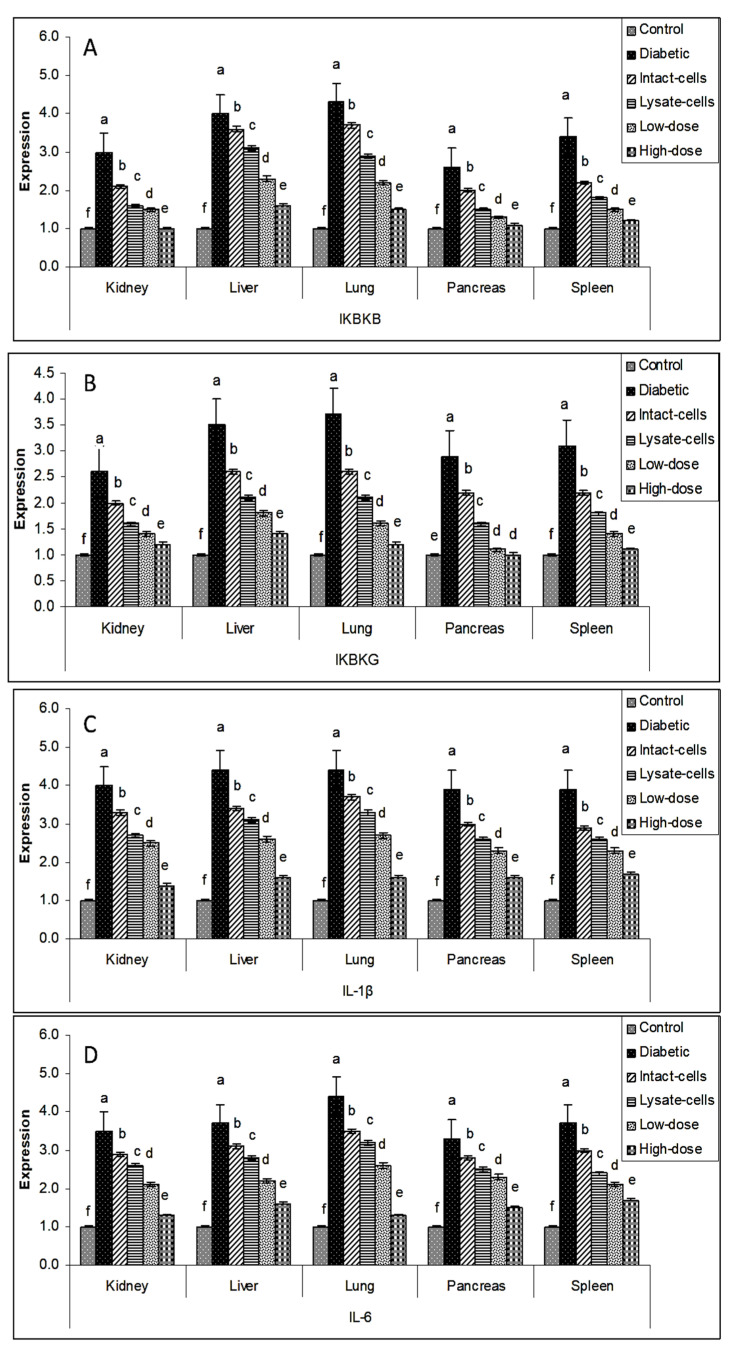
Impact of *L. plantarum* and *S. costus* on the regulation of (**A**) *IKBKB*, (**B**) *IKBKG*, (**C**) *IL-1β*, and (**D**) *IL-6* genes in the liver, spleen, pancreas, lung, and kidney tissues of treated and non-treated rats. The β-actin gene was used to normalize the comparative mRNA levels. ^a,b,c,d,e,f^: bars with different letters in the same tissue differ significantly (*p* < 0.05).

The manner of expression differed depending on the tissue. The overall tone of regulating the major target genes was downregulated in the treatment groups (intact cells, lysate cells, low dose, and high dose) compared to the diabetes group in various organ tissues (liver, spleen, pancreas, lung, and kidney). The use of a high dosage of *S. costus* (100 mg kg^−1^ BW) on all tested organs resulted in a significant drop in gene expression for all sought genes, and low dose, lysate cell, and intact cell treatments followed. Gene expression profiles of *IKBKB*, *IKBKG*, *IL-1β,* and *IL-6* genes in the liver, spleen, pancreas, lung, and kidney tissues exhibited high transcript levels in the diabetic groups compared to the control groups. In contrast, the different treated groups exhibited diminished levels of transcripts compared to the diabetic groups, as shown in Figure 5.

**Figure 6:**
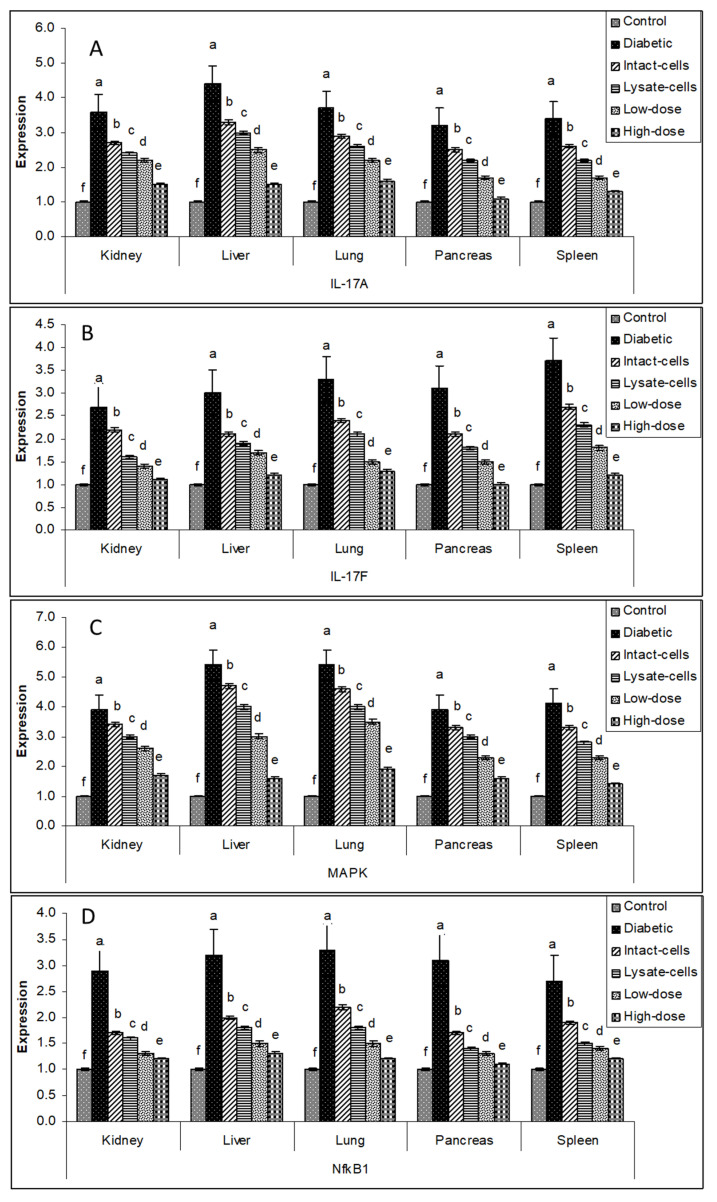
Impact of *L. plantarum* and *S. costus* on regulating (**A**) *IL-17A*, (**B**) *IL-17F*, (**C**) *Mitogen-activated protein kinase 1 (MAPK)*, and (**D**) *NFKB1* genes in the liver, spleen, pancreas, lung, and kidney tissues of treated and non-treated rats. The β-actin gene was used to normalize the comparative mRNA levels. ^a,b,c,d,e,f^: bars with different letters in the same tissue differ significantly (*p* < 0.05).

Similar results were investigated for *IL-17A*, *IL-17F*, *MAPK*, and *NFKB1* genes in the liver, spleen, pancreas, lung, and kidney tissues. There was negative regulation in the different treated groups compared to the diabetic groups indicated in Figure 6. 

**Figure 7:**
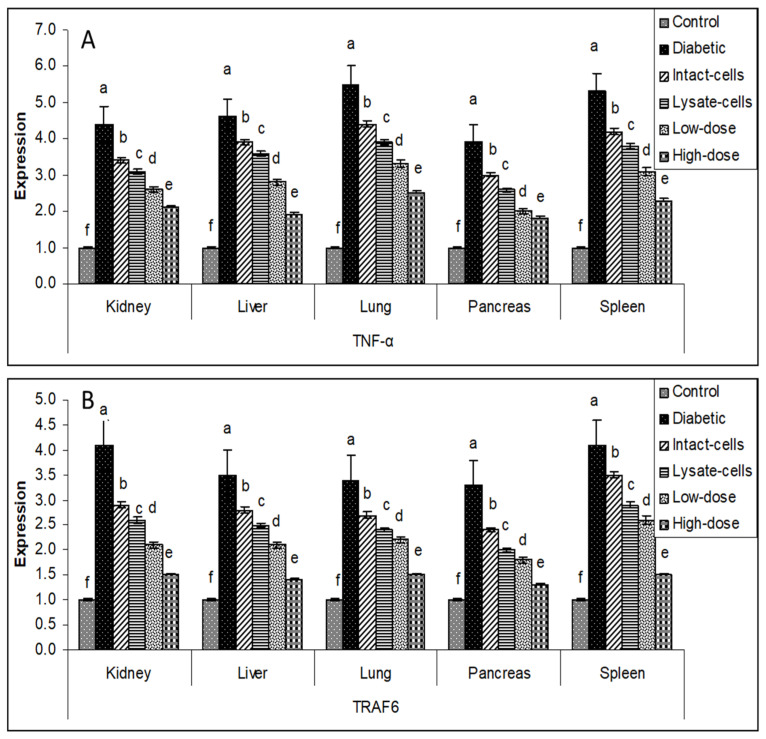
The impact of *L. plantarum* and *S. costus* on (**A**) *TNF-α* and (**B**) *TRAF6* gene regulation in the liver, spleen, pancreas, lung, and kidney tissues of treated and non-treated rats. The β-actin gene was used to normalize the comparative mRNA levels. ^a,b,c,d,e,f^: means with different letters in the same tissue differ significantly (*p* < 0.05).

Similarly, the group with the high dose of *S. costus* showed the lowest expression profile for *TNF-α* and *TRAF6* genes, as illustrated in Figure 7. 

### 3.7. Druglikeness and Toxicity Risk Assessment 

Data from the SwissSimilarity and SwissADME online tools for dehydrocostus lactone and its derivatives revealed that dehydrocostus lactone, caprolactone, methyl nonanoate ester, (3E)-3-[(phenylamino)methylidene]oxan-2-one, gamma-valerolactone, butyl acetate, and (3E)-3-[(phenylamino)methylidene]dihydrofuran-2(3H)-one each had two H-bond acceptors. In contrast, bigelovin, ethyl levulinate, and R-carvone had five, three, and one, respectively. Except for bigelovin, the LogP values of the ligands were less than 5. Table 7 shows that the methyl nonanoate ester had the lowest LogS value dehydrocostus lactone, whereas caprolactone had the highest value, followed by ethyl levulinate. Moreover, all ligands mentioned in Table 7 had a molecular weight of less than 500 Da. All ligands agreed with Lipinski’s criteria. In addition to immunotoxicity for dehydrocostus lactone and carcinogenicity for ethyl levulinate and butyl acetate, data from the ProTox-II online tool indicated that hepatotoxicity, mutagenicity, and cytotoxicity were all inactive. Bigelovin, caprolactone, methyl nonanoate ester, (3E)-3-[(phenylamino)methylidene] oxan-2-one, gamma-valerolactone (3E)-3-[(phenylamino)methylidene]dihydrofuran-2(3H)-one, and r-carvone demonstrated no hepatotoxicity, immunotoxicity, mutagenicity, cytotoxicity, and carcinogenicity, as shown in Table 8. 

### 3.8. Molecular Docking

**Figure 8:**
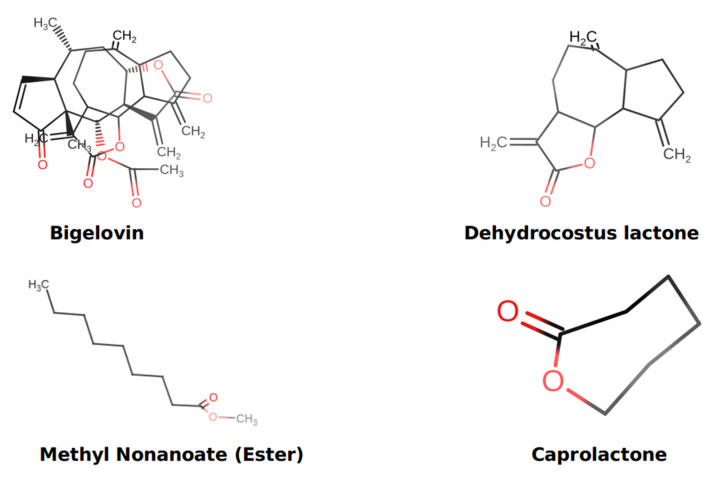
The 3D chemical structure of the selected ligands for molecular docking (dehydrocostus lactone, bigelovin, caprolactone, and methyl nonanoate ester. SMILES were retrieved from the PubChem database https://pubchem.ncbi.nlm.nih.gov accessed on 14 May 2023 and were drawn with the Marvin JS tool https://marvinjs-demo.chemaxon.com/latest/demo.html accessed on 14 May 2023.

Molecular docking was carried out with minor modifications using the described flexible docking approach [47]. The molecular docking investigation of the identified ligands with our target proteins was carried out using AutoDock Vina. PDBQT was an acronym for the protein data bank, partial charge, and atom type (using their previously created PDB files as inputs). The grid box was altered according to the active sites of each protein molecule (coordinates: x = 48.3895, y = 29.1055, z = −57.3299, sizes: x = 10.8403, y = 9.47439, z = 11.6949), and all bonds in the ligand were allowed to spin freely, resulting in a stiff receptor. After completing the molecular docking studies and forming 10 configurations for each protein–ligand combination for all ligands, text files containing the score data were created for manual comparison. The optimal docking site was chosen as the conformation with the lowest binding energy (BE, kcal/mol) and root-mean-square deviation (RMSD). However, an exhaustiveness of 10 was chosen for docking throughout this in silico experiment to obtain more precise and reliable results, and the number of modes was fixed at 10. Using the UCSF-Chimera, Maestro, and Discovery studio tools, the interaction between ligands and proteins was then produced, visualized, and analyzed. As shown in Figure 8, Marvin JS picked and visualized the 2D structure of four ligands with high-affinity ratings.

**Figure 9:**
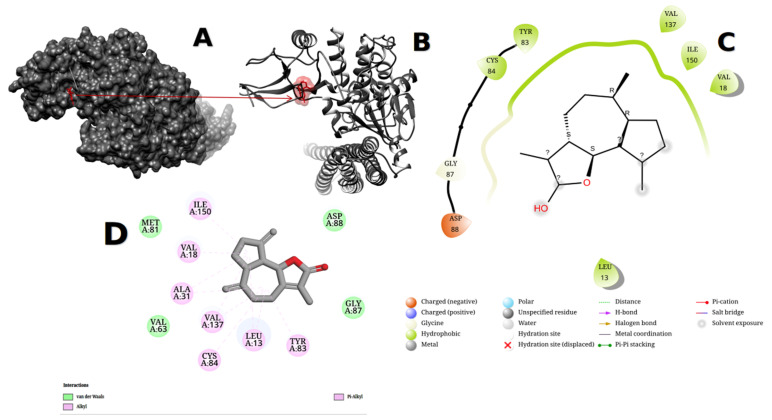
4KIK protein showed the pocket of ligand dehydrocostus lactone after docking using UCSF-Chimera version 1.17.1 and visualized it (**A**,**B**). The obtained affinity score was (−8.619 kcal/mol). The ligand–protein interaction was performed in Maestro software version 2022-4 and Discovery Studio version 2021 (**C**,**D**).

**Figure 10:**
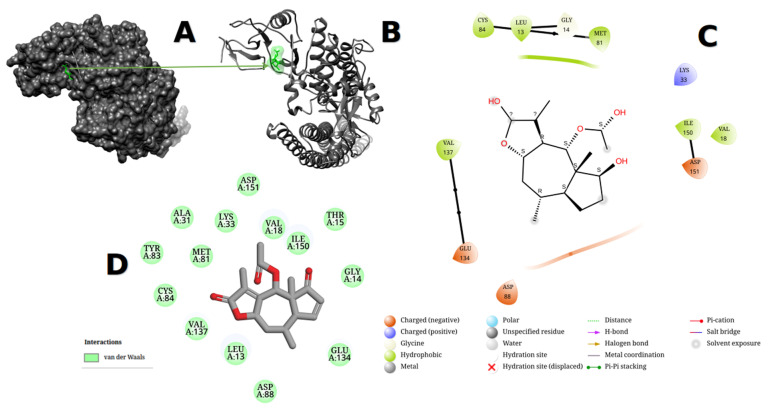
4KIK protein showed the pocket of ligand bigelovin after docking using UCSF-Chimera version 1.17.1 and visualized it (**A**,**B**). The obtained affinity score was (−7.882 kcal/mol). The ligand–protein interaction was performed in Maestro version 2022-4 and Discovery Studio version 2021 (**C**,**D**).

In our present in silico study, dehydrocostus lactone derivatives were docked against 4KIK using Auto Dock Vina. This study’s 4KIK was discovered in its 3D crystal structure; it was co-crystallized with a human IkB kinase beta with a resolution of 2.83 Å, had a sequence length of 677 amino acids, and had an inhibitor of nuclear factor kappa-B kinase subunit beta. Asp88, GLY87, CYS84, TYR83, VAL137, ILE150, and VAL18 surrounded 4KIK’s binding site with dehydrocostus lactone, as shown by Maestro software, while Asp88, GLY87, CYS84, TYR83, VAL137, ILE150, VAL18, ALA31, VAL63, and MET81 surrounded 4KIK’s binding site with dehydrocostus lactone, as shown by Discovery Studio. According to Maestro software, the binding site revealed charged (negative), glycine, and hydrophobic interactions. In contrast, Discovery Studio displayed van de Waals, alkyl, and Pi-Alkyl interactions, as shown in Figure 9C,D. The maximum binding energy was −8.619 kcal/mol for dehydrocostus lactone. This sparked the idea to use UCSF-Chimera, Maestro, and Discovery Studio software to compare their binding locations and interaction patterns with the target protein to the best hit control medicine (dehydrocostus lactone). Furthermore, at bond lengths of 3.72 Å, 3.03 Å, 3.78 Å, 3.92 Å, and 3.45 Å, dehydrocostus lactone was able to interact with amino acids inside the binding pouch of 4KIK, including LEU13, VAL18, TRY83, VAL137, and ILE150. The bond lengths were calculated using the PLIP online tool, available at https://plip-tool.biotec.tu-dresden.de/plip-web/plip/index accessed on 15 May 2023. Bigelovin bound to 4KIK via ASP88, ASP151, VAL18, ILE150, LYS33, MET81, GLY14, LEU13, CYS84, VAL137, GLU134, ALA31, and TRY83. According to Maestro software, the binding area exhibited charged (positive), charged (negative), glycine, and hydrophobic interactions, as shown in Figure 10. The conjugated energy of bigelovin was −7.882 kcal/mol. The chemical was discovered to fit precisely into the cavity of the binding site. Bigelovin also interacted with six amino acids inside 4KIK’s binding pouch, including VAL18, LYS33, ASP88, VAL137, ILE150, and ASP151, with bond lengths of 3.48 Å, 3.96 Å, 3.62 Å, 3.88 Å, 3.58 Å, and 3.76 Å. The bond lengths were calculated using the online PLIP tool [48].

**Figure 11:**
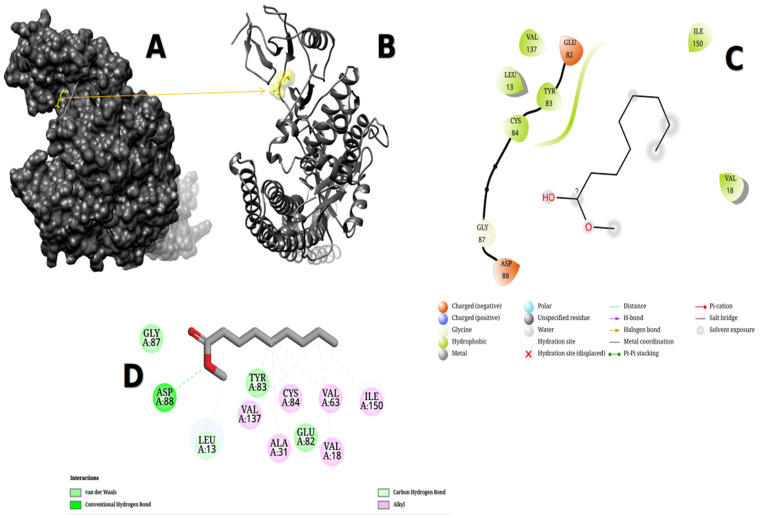
4KIK protein showed the pocket of caprolactone after docking using UCSF-Chimera version 1.17.1 and visualized it (**A**,**B**). The obtained affinity score was (−5.574 kcal/mol). The ligand–protein interaction was performed in Maestro version 2022-4 and Discovery Studio version 2021 (**C,D**).

Caprolactone, VAL18, ILE150, GLU82, VAL137, TRY83, LEU13, CYS84, GLY87, ASP88, ALA31, and VAL63 were shown by Maestro software, while VAL18, ILE150, GLU82, VAL137, TRY83, LEU13, CYS84, GLY87, ASP88, ALA31, and VAL63 were shown by Discovery Studio software. According to Maestro software, the binding site had charged (negative), glycine, and hydrophobic contacts. At the same time, Discovery Studio displays van de Waals, alkyl, conventional hydrogen bond, and carbon–hydrogen bond interactions, as seen in Figure 11. The binding energy of caprolactone was −5.574 kcal/mol. It was determined that the chemical fit into the identical cavity of the binding site. Caprolactone also interacted with five amino acids inside 4KIK’s binding pouch, including LEU13, VAL18, TRY83, VAL137, and ILE150, with bond lengths of 3.79 Å, 3.60 Å, 3.84 Å, 3.79 Å, and 3.63 Å, respectively, according to data retrieved from https://plip-tool.biotec.tu-dresden.de/plip-web/plip/index.html accessed on 15 May 2023.

**Figure 12:**
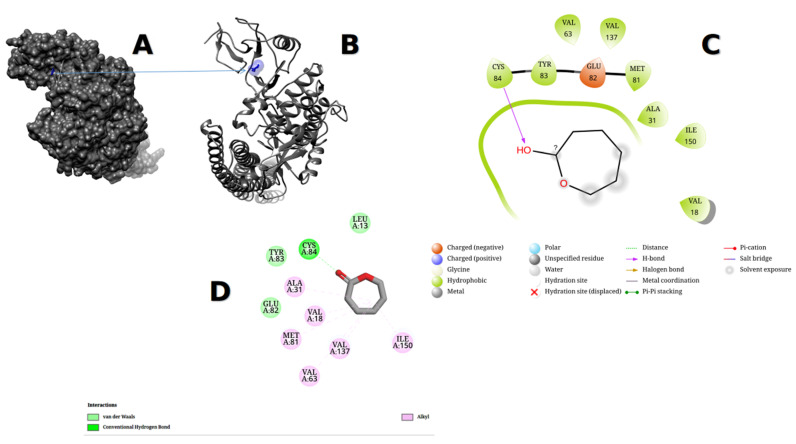
4KIK protein showed the pocket of the methyl nonanoate ester after docking using UCSF-Chimera version 1.17.1 and visualized it (**A**,**B**). The obtained affinity score was (−4.997 kcal/mol). The ligand–protein interaction was performed in Maestro version 2022-4 and Discovery Studio version 2021(**C**,**D**).

Maestro demonstrated a ring made of VAL18, ILE150, ALA31, MET91, VAL137, VAL63, GLU82, TRY83, CYS84, and LEU13, whereas Discovery Studio demonstrated a ring made of VAL18, ILE150, ALA31, MET91, VAL137, VAL63, GLU82, TRY83, CYS84, and LEU13. As seen in Figure 12, Maestro recognized charged (negative) and hydrophobic connections, whereas Discovery Studio detected de van Waals, alkyl, and conventional hydrogen bond interactions. A total of −4.997 kcal/mol was the enchained force of the methyl nonanoate ester. The chemical was found to fit into the exact cavity of the binding site. Furthermore, with bond lengths of 3.54 Å, 3.83 Å, 3.88 Å, 3.96 Å, 3.59 Å, and 1.98 Å, VAL18, ALA31, VAL63, MET81, ILE150, and CYS84 interacted with the methyl nonanoate ester inside the binding pouch of 4KIK.

**Figure 13:**
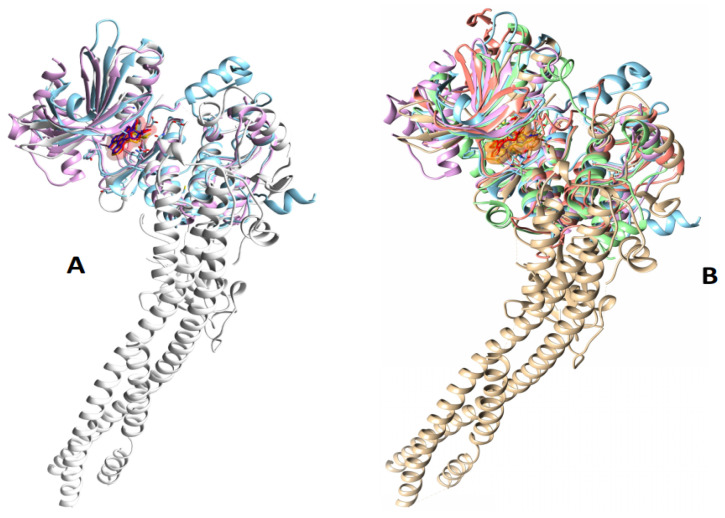
(**A**) The three proteins (4KIK, 4WSQ, and 5M5A) and (**B**) five proteins (4KIK, 4WSQ, 5M5A, 3EQF, and 1ROP) kinases were compared with the same ligand KSA (448239) in MatchMaker, UCSF-Chimera version 1.17.1.

The five kinase protein receptors were found in the PDB data bank, which may be found at https://www.rcsb.org/ accessed on 10 December 2022. The files should then be saved as PDB. The RMSD values for 4KIK, 3EQF, 1ROP, 4WSQ, and 5M5A were 0.000 Å, 2.171 Å, 2.699 Å, 25.503 Å, and 1.365 Å, respectively, using PyMOL software version 2.5.0. UCSF-Chimera was used to produce the PDB files. In five protein kinases, the dehydrocostus lactone ligand was the ligand (4KIK, 4WSQ, 5M5A, 3EQF, and 1ROP). A matchmaker was used for pairing the five target proteins. The MatchMaker analysis found the same pocket of interacting sites, as seen in Figure 13.

### 3.9. Molecular Dynamic Simulation

Algorithm performance and accuracy in the molecular dynamic (MD) simulation helps decode and predict compound stability. This procedure was critical for comprehending molecular and atomic-level alterations and assessing the stability of protein–ligand complex interactions. Structure-based small molecule design approaches, such as molecular docking and virtual screening, have resulted in the development of several medicines. MD simulations may also be used to assess the dynamic activity and stability of the ligand concerning the protein.

**Figure 14:**
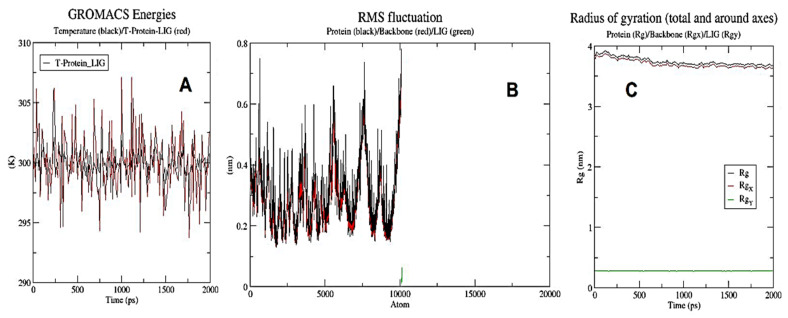
GROMACS energies chart displayed temperature with a black curve, while T-Protein-LIG was a red color curve. (**A**) RMS fluctuation chart displayed proteins with a black curve, a backbone with a red curve, and ligands with a green curve (**B**). The radius of gyration (total and around axes) chart showed proteins with a black curve, a backbone with a red curve, and a ligand with a green curve, respectively (**C**). The previous GROMACS analysis was performed on GROMACS version 2022.4 for 4KIK protein with dehydrocostus lactone and was visualized with Grace version 5.1.25.

**Figure 15:**
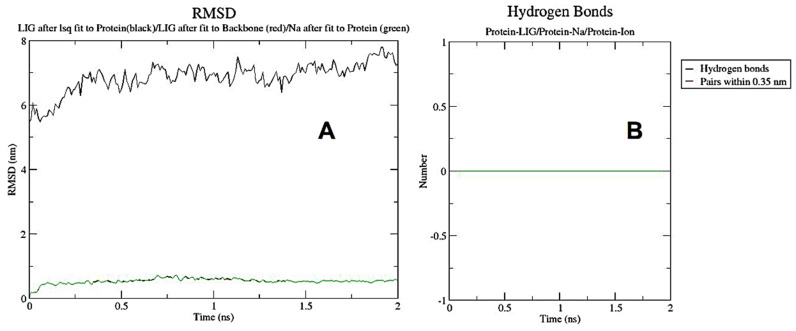
RMSD chart showed ligand–protein with a black curve, ligand–backbone with a red curve, and Na–protein with a green curve, respectively (**A**). The hydrogen bonds chart displayed protein with ligands, Na, and ions at the same level in green (**B**). The previous GROMACS analysis was performed on GROMACS version 2022.4 for 4KIK protein with dehydrocostus lactone and was visualized with Grace version 5.1.25.

Figure 14A,B displayed the RMSF (ranging from 0.15 to 0.8 nm for proteins and 0.0 to 0.056 nm for ligands) and GROMACS energies (ranging from 395 k to 305 k). In contrast, the variance in a protein’s backbone during 100 ns simulations was calculated using the root-mean-square deviation (RMSD) value. As the temperature rises, the complex goes through a period of volatility before stabilizing. The RMSD for human IkB kinase beta in combination with dehydrocostus lactone ranged between 5.5 and 7.2 nm over roughly 2 ns, with ligand changes ranging from 0.0 to 0.6 nm (Figure 15A). Figure 15B depicted hydrogen bonds between proteins and ligands, proteins and Na, and proteins and ions. Finally, Figure 14C revealed how the ligands and protein’s radii of gyration were successfully coupled.

## 4. Discussion

Diabetes alters the patient’s body in a variety of ways. As the malady progresses, the patient’s condition may deteriorate; some of these changes may result in serious health consequences or organ dysfunction [49]. Alloxan data revealed several diabetes issues compared to the control group, including body weight loss and a decrease in daily weight gain. Dehydration, as well as fat and protein catabolism, may contribute to diabetes-related body weight loss. Catabolic processes that cause muscle atrophy could explain why diabetic rats gain less weight [50].

Cortisol production was increased due to alloxan or stress, resulting in increased blood glucose from cortisol secretion and decreased weight for people with diabetes [51]. Additionally, an oxygen radical is an active oxygen atom without an electron that becomes destabilized. It seeks its missing electron by attacking any molecule within the cell, typically beginning with the cell membrane and ending with a piece of DNA [52]. Alloxan increases oxidative stress indicators and reduces natural antioxidant enzymes. The data showed a considerable rise in MDA and GSSG and a noteworthy decrease in GSH, all of which were insulin secretion hinders, leading to insulin-dependent diabetes mellitus. These two effects were because of its electron and chemical properties, such as its alkylating potency and NO donation capabilities [53].

High blood glucose and low insulin levels can increase cholesterol, TGs, and LDL and decrease HDL levels. Research findings suggest elevated cholesterol levels were detrimental to the liver and muscle tissues. This was in line with our findings from histological studies, which demonstrated significant disruption of haptic architecture in rats treated with alloxan. We detected generalized (>75% of the tissue) microvesicular and macrovesicular fatty degeneration of hepatocytes, variable degrees of dilation and congestion of hepatic sinusoids, and increased connective tissue between the hepatic lobules, as well as interlobular and periportal steatohepatitis. These findings were consistent with two previous studies using in vivo rat models [45,54]. Our data from both biochemical and histological studies indicated that the detected hypercholesterolemia induced cholesterol buildup in hepatocytes with subsequent fatty degeneration. Furthermore, cholesterol accumulation in liver cells promotes steatohepatitis through inflammatory cell infiltration and fibrosis induction [55,56]. Importantly, the TGs buildup in hepatocytes was previously linked with insulin resistance, putting the liver at risk of additional damage [57]. Additionally, alloxan induces the liver’s conversion of fatty acids to cholesterol and increases triglyceride formation, released into the bloodstream as lipoproteins [58]. Hence, the liver’s stellate cells may be stimulated by oxidative stress and steatohepatitis, which are detected in this study, leading to fibrosis.

Probiotics can reduce cholesterol levels in hamsters that eat mushrooms [59]. The current study showed that *L. plantarum* administered as a cell lysate or intact cells exhibited some degree of antioxidant and antihyperlipidemic effects compared to alloxan-induced diabetic rats. This was consistent with Werning et al. [60], who reported the antioxidant and DNA-protecting effects of the same bacteria using proteomic analysis. Our results were also corroborated by Pisosch et al. [61], who documented the lipid metabolism regulatory effect of *L. plantarum*. Upon further assessment of *L. plantarum* effects on histological aberration induced by alloxan, our results showed minimal to mild improvement of hepatocytes with cell lysate and intact cells, respectively. Our histological study revealed decreased micro and macrovesicular steatosis in hepatocytes, reduced inflammatory infiltrates, and connective tissue between hepatic lobules compared to diabetic rats. Our findings showed that *L. plantarum* intact cells had superior hepatoprotective properties compared to cell lysate. These findings can be justified because LAB improves food nutrient value while providing health advantages such as reducing the risk of infection, influencing immunity, preventing cancer, and regulating glucose serum levels [62]. Strengthening the body’s immune system can promote antioxidants, such as GSH, which act as cell membrane protectors and, as a result, reduce MDA production because of decreased cell destruction [63].

Our results demonstrated that *S. costus* administration in both low and high doses ameliorated the alloxan-induced oxidative stress in the liver. After the administration of *S. costus*, rats showed decreased levels of MDA, decreased GGSG activity, and increased GSH activity compared to diabetic rats. This agrees with other studies showing that *S. costus* decreases MDA and NO levels and increases GSH-Px and SOD activities [64]. Notably, the reduced MDA level after S. costus administration indicates diminished lipid peroxidation of hepatocyte cell membranes, contributing to hepatoprotection. Based on the lipid profiles of the present study, *S. costus* significantly decreased TC, TG, and LDL and increased HDL compared to diabetic rats. This was also supported by our histological analyses, which revealed a marked improvement in the histological architecture of the liver compared to alloxan-treated rats. A marked reduction in the large steatotic vacuoles with only minimal microvesicular vacuoles was detected in this group. These results agree with previous studies demonstrating costus’ hypolipidemic and anti-obesity effects using different in vivo rat models [57,65,66]. One can surmise that the hepatoprotective effect of *S. costus* can be attributed to its antihyperlipidemic and antioxidant activity.

Nuclear factor-kappa-B (NF-_K_B) was a transcription factor used in regulating inflammatory reactions and controlling the innate immune system [67]. Recent research has revealed the importance of NFB and I-kappaB-kinase (IKK) in developing insulin resistance and type 2 diabetes mellitus [68]. IKK was in charge of the phosphorylation and destruction of the inhibitor-kappaB complex, which allows NF-_K_B to be activated. TNF-α and IL1β pro-inflammatory cytokines activate NF-_K_B via conventional receptor-mediated pathways. TLRs (toll-like receptors) were important immune response regulators [69]. Thus, TLR4, a component of the bacterial lipopolysaccharide receptor, serves as a crucial link between innate and adaptive immunity. It also activates NF-_K_B, generating cytokines such as TNF-α and IL1β [70], as shown in Supplementary Materials Files S1–S3.

The movement of white blood cells from the circulation to the tissues characterizes inflammatory events. These activated immune cells and inflammations have lately been linked to the development of type 2 diabetes mellitus, and they appear to play a key role in modulating insulin resistance. In animal studies, the absence of IKK in immune cells completely protects against insulin resistance. Furthermore, nutrients may work through pathogen-sensing systems, such as TLRs, causing metabolically or nutritionally driven inflammatory responses [36], as shown in Supplementary Materials Files S1–S3.

Data on gene expression support the concept that metabolic syndrome causes generalized systemic inflammation mediated by the innate immune system [37]. As the pre-disease condition evolves to type 2 diabetes, this inflammation remains. Gene expression in the liver, spleen, pancreas, lung, and kidney organ tissues in type 2 diabetes identifies additional immune processes that may underpin this disease phenotype; however, qRT-PCR analysis of these genes in independent cohorts suggests that differential expression in type 2 diabetes may overlap to a greater extent than was evident by qRT-PCR analysis [38]. This finding was consistent with the overlap in biological processes and risk factors that underpin disease states. There was some difference between the control, diabetic, and treated diabetic rat groups; for example, *IKBKB*, *IKBKG*, *NfkB1*, *IL-17A*, *IL-6*, *IL-17F*, *IL-1β*, *TNF-α*, *TRAF6*, and *MAPK* expression appeared to be upregulated in the diabetic rats compared to the control rats. As demonstrated in Figure 6, Figure 7 and Figure 8, the gene regulation of the target genes was gradually downregulated in the studied organ tissues in intact cells, lysate cells, and low-dose treatments.

Given that conformational changes during the ligand–receptor binding process had a significant impact on the docking effect, nine small compounds were chosen for multiple docking, and the best pose was chosen for MD simulation experiments; all compounds had larger conformational changes of residues during the binding process, whereas ten compounds were chosen for semiflexible docking experiments; all compounds had larger conformational changes of ligands during the binding process. All crystal structure compound files for the experiments were obtained from the RCSB database (https://www.rcsb.org). AutoDock vina software was used to prepare the files for the numerous docking trials. Preparing files for the human IkB kinase beta receptor (4KIK) in the flexible docking and MD simulation experiments and preparing files for the dehydrocostus lactone derivatives in the semiflexible docking experiments were illustrated in Figure 11, Figure 12, Figure 13, Figure 14 and Figure 15.

In the current study, *S. costus*, due to its hematopoietic properties, helps to normalize alloxan dysfunction. After lactone administration, an alloxan-induced acute inflammatory response helps restore the body’s normal homeostatic state. Lactone reduces inflammation by inhibiting cyclooxygenase and inducible nitric oxide synthase [71]. The results also demonstrated decreased glucose levels and improved glucose tolerance due to enhanced digestion resistance. Finally, the results confirmed that probiotic cell lysate was less successful in reducing symptoms than *S. costus* as a botanical source.

## 5. Conclusions

Examining histological and biochemical data, the high dose of *S. costus* prepared from a botanical source showed potential for treating diabetes and other complications attributed to ameliorating body weight, lipid profile, oxidative stress, insulin, and glucose levels. A low dose of *S. costus*, cell lysate of *L. plantarum*, and intact cells of *L. plantarum* treatments, respectively, showed less downregulation of *IKBKB*, *IKBKG*, *NfkB1*, *IL-17A*, *IL-6*, *IL-17F*, *IL-1β*, *TNF-α*, *TRAF6*, and *MAPK* genes compared with a high dose of *S. costus* treatment in diabetic rats. Molecular docking and MD simulation data confirmed the interaction between human IkB kinase beta protein and dehydrocostus lactone as a drug. The target genes are important in regulating type 2 diabetes mellitus signaling, lipid and atherosclerosis signaling, NF-κB signaling, and IL-17 signaling pathways.

## Figures and Tables

**Table 2:** The effect of *L. plantarum* and *S. costus* doses depends on the body weight of non-diabetic and alloxan-induced diabetic rats.

Groups	Parameters		
	Initial Body Weight (g)	Final Body Weight (g)	Body Weight Gain (g)
Control	154 ± 5.85 ^a^	291 ± 2.36 ^a^	137 ± 5.74 ^a^
Diabetic	159 ± 11.23 ^a^	153 ± 2.12 ^e^	−6 ± 12.15 ^e^
Diabetic + intact cells	161 ± 5.29 ^a^	203 ± 1.51 ^d^	42 ± 4.25 ^d^
Diabetic + lysate cells	166 ± 9.81 ^a^	235 ± 3.11 ^c^	69 ± 7.88 ^c^
Diabetic + low dose	168 ± 7.78 ^a^	237 ± 2.16 ^c^	69 ± 8.91 ^c^
Diabetic + high dose	165 ± 2.88 ^a^	255 ± 3.00 ^b^	91 ± 2.87 ^b^

Lysate cells: *L. plantarum* lysate cells, intact cells: *L. plantarum* intact cells, low dose: *S. costus* low dose, high dose: *S. costus* high dose, ^a,b,c,d,e^: mean having different superscript letters in the same row differ significantly (*p* < 0.05).

**Table 3:** The effect of *L. plantarum* and *S. costus* doses depends on the oxidative stress marker of non-diabetic and alloxan-induced diabetic rats.

Groups	Parameters		
	MDA nmol/mL	GSH µmol/mL	GSSG µmol/mL
Control	83.9 ± 3.49 ^e^	3.6 ± 0.14 ^a^	0.74 ± 0.03 ^d^
Diabetic	195.8 ± 8.42 ^a^	2.0 ± 0.08 ^b^	1.82 ± 0.06 ^a^
Diabetic + intact cells	157.1 ± 6.65 ^b^	2.5 ± 0.11 ^b^	1.74 ± 0.08 ^a^
Diabetic + lysate cells	149.4 ± 6.05 ^b^	2.5 ± 0.11 ^b^	1.47 ± 0.06 ^b^
Diabetic + low dose	130.3 ± 5.55 ^c^	3.0 ± 0.12 ^a^	1.41 ± 0.05 ^b^
Diabetic + high dose	106.9 ± 4.26 ^d^	3.6 ± 0.15 ^a^	1.11 ± 0.04 ^c^

Lysate cells: *L. plantarum* lysate cells, intact cells: *L. plantarum* intact cells, low dose: *S. costus* low dose, high dose: *S. costus* high dose, ^a,b,c,d,e^: means with different superscript letters in the same row differ significantly (*p* < 0.05).

**Table 4:** The effect of *L. plantarum and S. costus* doses depends on the lipid profile of non-diabetic and alloxan-induced diabetic rats.

Groups	Parameters			
	TG mg dL^−1^	TC mg dL^−1^	HDL mg dL^−1^	LDL mg dL^−1^
Control	96.6 ± 4.03 ^d^	83.8 ± 3.033 ^e^	34.5 ± 1.29 ^a^	45.7 ± 21 ^f^
Diabetic	231.1 ± 10.02 ^a^	215.9 ± 7.88 ^a^	19.5 ± 0.79 ^d^	162.2 ± 6.98 ^a^
Diabetic + intact cells	230.6 ± 8.99 ^a^	209.4 ± 8.54 ^a^	24.3 ± 0.94 ^c^	150.7 ± 6.02 ^b^
Diabetic + lysate cells	184.7 ± 8.17 ^b^	186.4 ± 7.72^4 b^	23.2 ± 0.88 ^c^	134.2 ± 4.87 ^c^
Diabetic + low dose	165.2 ± 6.54 ^c^	165.1 ± 6.07 ^c^	31.4 ± 1.28 ^b^	120 ± 4.93 ^d^
Diabetic + high dose	158.4 ± 5.67 ^c^	132.4 ± 5.59 ^d^	33.3 ± 1.32 ^a^	83.2 ± 3.51 ^e^

Lysate cells: *L. plantarum* lysate cells, intact cells: *L. plantarum* intact cells, low dose: *S. costus* low dose, high dose: *S. costus* high dose, ^a,b,c,d,e,f^: means with different superscript letters in the same row differ significantly (*p* < 0.05).

**Table 5:** The effect of *L. plantarum* and *S. costus* doses depends on the glucose, insulin, and HOMA-IR in non-diabetic and alloxan-induced diabetic rats.

Groups	Parameters		
	Glucose mg dL^−1^	Insulin pmol L^−1^	HOMA-IR
Control	92.4 ± 3.44 ^f^	57.5 ± 2.14 ^a^	13.1 ± 0.1 ^c^
Diabetic	233.5 ± 8.81 ^a^	28.8 ± 1.12 ^d^	16.6 ± 0.12 ^b^
Diabetic + intact cells	221.4 ± 8.81 ^b^	31.9 ± 1.28 ^cd^	17.4 ± 0.29 ^b^
Diabetic + lysate cells	193.3 ± 7.31 ^c^	35.8 ± 1.37 ^c^	17.1 ± 0.35 ^b^
Diabetic + low dose	57.5 ± 6.83 ^d^	44.9 ± 1.96 ^b^	18.4 ± 0.39 ^a^
Diabetic + high dose	152.9 ± 6.54 ^e^	47.2 ± 1.84 ^b^	17.8 ± 0.36 ^a^

Lysate cells: *L. plantarum* lysate cells, intact cells: *L. plantarum* intact cells, low dose: *S. costus* low dose, high dose: *S. costus* high dose, ^a,b,c,d,e,f^: means with different superscript letters in the same row differ significantly (*p* < 0.05).

**Table 6:** Histological scoring of the H&E-stained sections based on an Ishak-modified HAI system in the different study groups.

Groups	Histological Score	Degenerative Changes	Type of Pathological Changes
Control	1	No abnormalities	None
Diabetic	5	Extensive and marked	Fatty degeneration (microvesicular and macrovesicular steatosis), fibrosis, inflammation, and blood vessel congestion.
Diabetic + intact cells	4	Severe	Moderate fatty degeneration (microvesicular and macrovesicular steatosis), inflammation, and blood vessel congestion.
Diabetic + lysate cells	3	Moderate	Minimal fatty degeneration (microvesicular and macrovesicular steatosis), inflammation, and blood vessel congestion.
Diabetic + low dose	3	Moderate	Minimal fatty degeneration (microvesicular and macrovesicular steatosis), inflammation, and congested hepatic sinusoids.
Diabetic + high dose	2	Mild	Minimal microvesicular steatosis, few congested hepatic sinusoids.

Lysate cells: *L. plantarum* lysate cells, intact cells: *L. plantarum* intact cells, low dose: *S. costus* low dose, high dose: *S. costus* high dose.

**Table 7:** The SwissADME was used to predict the drug-likeness of dehydrocostus lactone and its isomers based on Swiss similarity using Lipinski’s rule.

Compound	Formula	Similarity Score	MW (<500 Da)	H-Bond Acceptors (<10)	H-Bond Donors (<5)	Consensus Log P (<5)	Lipinski Violations	Ali Log S
Dehydrocostus lactone	C_15_H_18_O_2_	1	230.3	2	0	2.98	0	−2.82
Bigelovin	C_17_H_20_O_5_	0.943	304.34	5	0	1.89	0	−2.25
Caprolactone	C_6_H_10_O_2_	0.90	114.14	2	0	1.08	0	−0.11
Methyl nonanoate ester	C_10_H_20_O_2_	0.816	172.26	2	0	3.13	0	−4.59
(3E)-3-[(phenylamino)methylidene]oxan-2-one	C_12_H_13_NO_2_	0.748	203.24	2	1	2.14	0	−2.74
Gamma-valerolactone	C_5_H_8_O_2_	0.739	100.12	2	0	0.91	0	−0.74
Ethyl levulinate	C_7_H_12_O_3_	0.735	144.17	3	0	0.81	0	−0.53
Butyl acetate	C_6_H_12_O_2_	0.698	116.16	2	0	1.53	0	−1.95
(3E)-3-[(phenylamino)methylidene]dihydrofuran-2(3H)-one	C_11_H_11_NO_2_	0.646	189.21	2	1	1.84	0	−2.37
R-carvone	C_10_H_14_O	0.583	150.22	1	0	2.44	0	−2.72

The retrieved data are from http://www.swisssimilarity.ch/, accessed on 14 May 2023. Bioactive compounds, library compounds (LigandExpo), and combined 2D and 3D structure databases.

**Table 8:** Toxicity risk assessment employing the ProTox-II tool.

Compound	Hepatotoxicity (P)	Carcinogenicity (P)	Immunotoxicity (P)	Mutagenicity (P)	Cytotoxicity (P)	LD_50_ mg kg^−1^
Dehydrocostus lactone	0.59 i	0.61 i	0.94 a	0.72 i	0.76 i	1330
Bigelovin	0.61 i	0.50 i	0.99 i	0.78 i	0.81 i	125
Caprolactone	0.72 i	0.61 i	0.98 i	0.96 i	0.66 i	4290
Methyl nonanoate ester	0.58 i	0.55 i	0.99 i	0.98 i	0.73 i	5000
(3E)-3-[(phenylamino)methylidene]oxan-2-one	0.62 i	0.50 i	0.99 i	0.72 i	0.64 i	1800
Gamma-valerolactone	0.66 i	0.54 i	0.98 i	0.91i	0.66 i	8800
Ethyl levulinate	0.78 i	0.59 a	0.99 i	0.96 i	0.82 i	5000
Butyl acetate	0.77 i	0.57 a	0.98 i	0.97 i	0.81 i	6000
(3E)-3-[(phenylamino)methylidene]dihydrofuran-2(3H)-one	0.58 i	0.53 i	0.99 i	0.69 i	0.67 i	2761
R-carvone	0.65 i	0.83 i	0.99 i	0.97 i	0.80 i	1640

P: probability, a: active, i: inactive, https://tox-new.charite.de/protox_II/index.php?site=compound_input, accessed on 14 May 2023.

## Data Availability

Data and materials are available upon request from the corresponding author. Data is not publicly available due to privacy.

## Supplementary Materials

The following supporting information can be downloaded at: https://www.mdpi.com/article/10.3390/metabo13060764/s1. Supplementary Materials Files S1–S3.
