# Supplementary figures and images for: Lactiplantibacillus plantarum and Saussurea costus as Therapeutic Agents against a Diabetic Rat Model—Approaches to Investigate Pharmacophore Modeling of Human IkB Kinase and Molecular Interaction with Dehydrocostus Lactone of Saussurea costus
Source: Metabolites. 2023 Jun 19;13(6):764. doi: 10.3390/metabo13060764 (PMC10302201; doi:10.3390/metabo13060764)

Supplementary File S1 (S1)

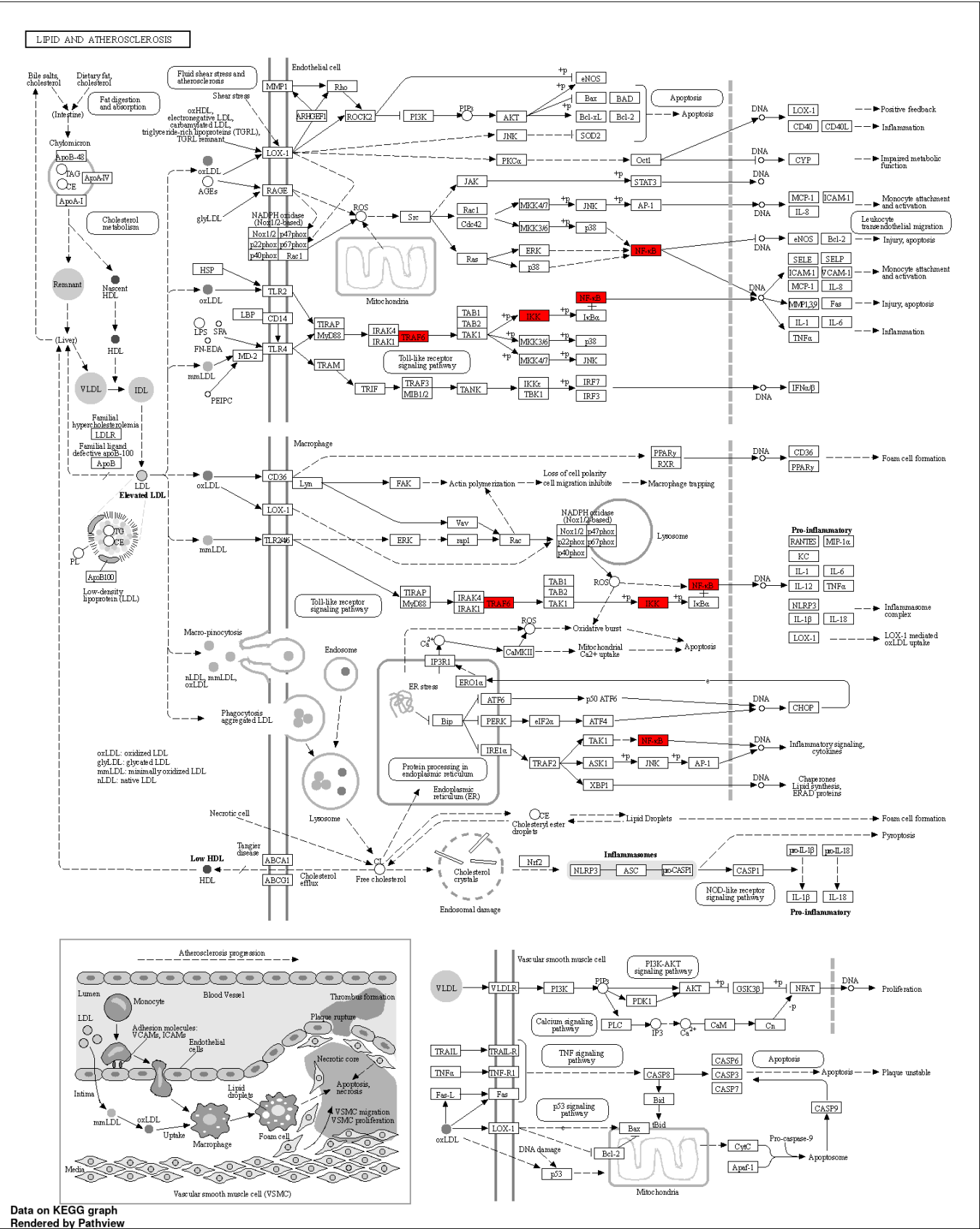

## Supplementary file 2 (S2)

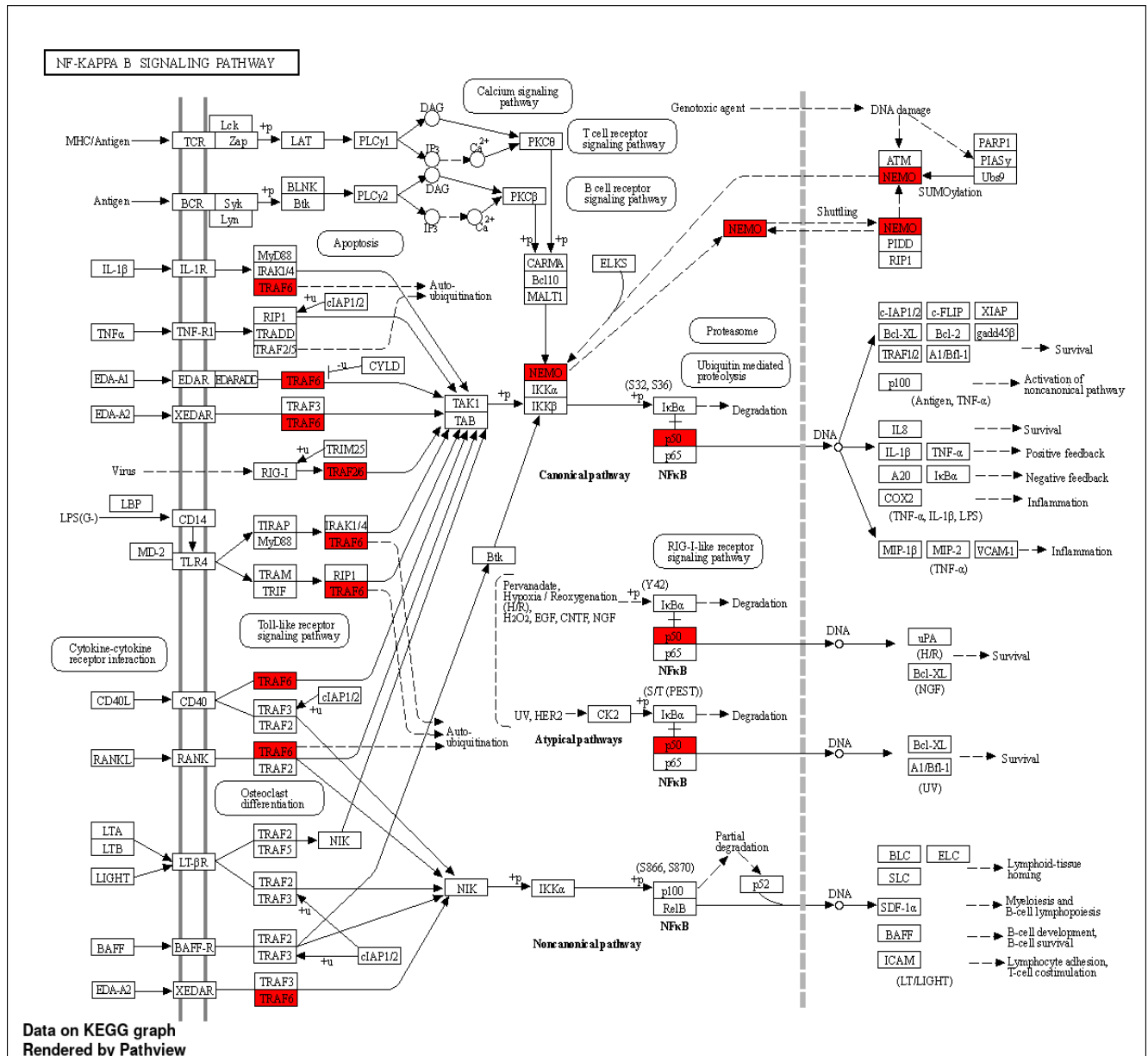

Supplementary file 3 (S3)

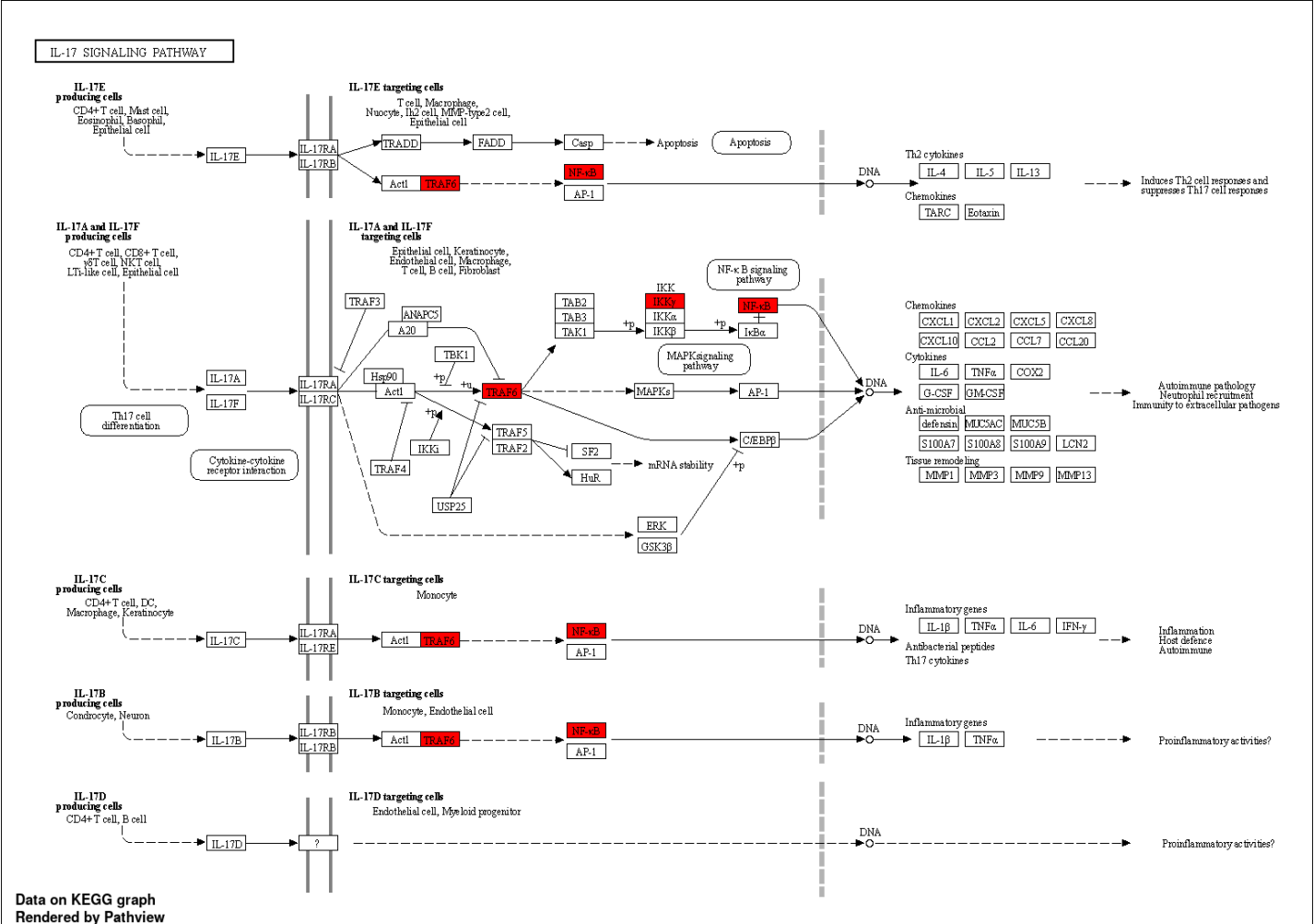

Supplement: Supplementary file 1 [file metabolites-13-00764-s001.zip › metabolites-2259433-supplementary.pdf]
